# A Case of Accidental Hæmorrhage

**Published:** 1868-04-15

**Authors:** De Laskie Miller

**Affiliations:** Prof. Obstetrics, Diseases of Women, Etc., Rush Medical College


### A CASE OF ACCIDENTAL HÆMORRHAGE.

BY DE LASKIE MILLER, M.D., PROF. OBSTETRICS, DISEASES OF WOMEN, ETC., RUSH MEDICAL COLLEGE.

Mrs. L., aged 40 years, the mother of six children, the last confinement, January 1, 1866, had aborted twice. Was advanced about seven months in her ninth pregnancy, which had been unattended by any unusual symptoms, when, on January 25th ult., at about 3 o'clock, P.M., she was suddenly attacked with fainting — a sense of distension of the abdomen, great anxiety, but without pain.

I saw her at 5 o'clock, about two hours after the attack; found her in an alarming condition, and presenting the following appearance and symptoms: remarkable pallor of the face; lips apparently bloodless; pulse hardly perceptible at the wrist; surface cold, frequently yawning; abdomen greatly enlarged and tense, with no resonance on percussion; slight hæmorrhagic discharge from the vulva; os uteri dilated sufficiently to admit the extremity of the index finger only, and not dilatable with moderate force.

The conclusion at once reached was, that the symptoms were due to internal hæmorrhage, the result of placental rupture or detachment.

On inquiry learned that no accident had happened which would be likely to cause the separation of the placenta from its attachment with the uterus. The only circumstance to which the detachment could be attributed was inordinate laughter, at dinner, between one and two o'clock, P.M.

The danger appearing imminent, by my order the foot of the bed was elevated about eight or ten inches. Administered a full dose of the *Fl. Ext. of Ergot.* Applied uniform pressure over the abdomen; introduced the colpeurynter and distended the upper part of the vagina forcibly, to dilate the os uteri, and excite uterine contractions, but dilatation to a slight degree only was effected, and no labor pains were induced. I then withdrew the colpeurynter, and passed a catheter through the membranes. As the liquor amnii flowed away, slight uterine contractions could be detected. The dilatation of the os uteri was accelerated by mechanical force, applied by inserting and separating the fingers, not having a more efficient "dilator" at my command. The os yielded slowly; when it had reached about the size of a crown piece, coagulated blood began to escape in great quantity. Without the means of estimating the quantity accurately, I can only say, it was sufficient to account for the prostration, fainting, etc. With this discharge the abdomen diminished in size rapidly, contractions increased in force slightly, which brought the child within reach of the finger, when the presenting part was found to be the right shoulder. Notwithstanding the great loss of blood and consequent exhaustion of the patient, the os uteri was not sufficiently dilated or dilatable to admit the hand, for the purpose of version and prompt delivery. As delay would surely increase the danger, the blunt hook was passed over the pelvic extremity of the trunk, and sufficient traction made to bring this part down, and the delivery of the fœtus was effected without delay. The placenta followed immediately, and presented evidences of having been separated for some time. *Ergot* was repeated, and a binder firmly applied; stimulants were administered; the head kept low. By careful management the patient recovered slowly but perfectly.

This case presents several points of interest:

1st. The slight cause of the accident, viz.: a fit of laughter. I give this as the cause, for it could be attributed to no other. The patient was the wife of one of our most wealthy citizens, and not subject to the casualties incident to laborious pursuits.

2nd. The sudden appearance of dangerous symptoms: characteristic of loss of blood.

3rd. The importance of perforating the membranes, in cases like this, where delay increases the danger, and over distension prevents uterine contractions.

4th. The utility of the blunt hook in transverse presentations like this, in effecting version and prompt delivery, when the hand can not be introduced sufficiently to effect the object.

5th. The difficulty in effecting dilatation of the os uteri, so unusual in cases of extreme exhaustion from loss of blood.

